# Application of hydrolyzed proteins of animal origin in processed meat

**DOI:** 10.1002/fsn3.289

**Published:** 2015-10-06

**Authors:** Lene Meinert, Eva Honnens de Lichtenberg Broge, Camilla Bejerholm, Kirsten Jensen

**Affiliations:** ^1^ Danish Meat Research InstituteGregersensvej 92630TaastrupDenmark

**Keywords:** Bioactivity, by‐products, hydrolysates, slaughterhouse side streams

## Abstract

With increasing consumer interest in functional foods, proteins from slaughterhouse side streams can offer interesting application opportunities in this respect. Worldwide, increasing numbers of people are suffering from hypertension and protein deficiency. Hydrolyzed proteins of animal origin may show ACE‐inhibitory activity, which is central to the treatment of hypertension. Furthermore, the protein content of, for example, meat products increases markedly through the addition of hydrolyzed proteins, and these protein‐rich products are of interest to those suffering from protein deficiency. Through a series of analyses, six selected hydrolysates were analyzed for their application potential in the Danish meat product saveloy. Hydrolyzed pig rectum and bovine diaphragm showed the highest ACE‐inhibitory activities, and these activities were maintained in the processed saveloys. The ACE‐inhibitory activities could not readily be explained by the amino acid profile. The content of N‐compounds in the saveloys increased with increasing addition of hydrolysate, with little difference between the added hydrolysates. A sensory panel assessed the saveloys with added porcine rectum (8%), bovine diaphragm (8%), and bovine heart (4% and 8%) as having the strongest off‐flavors (chemical flavor). No increase in salty taste resulting from the addition of hydrolysates was detected in the saveloys. Finally, the consumers found the saveloys too mild in flavor and recommended the addition of more spices.

## Introduction

Consumers increasingly understand that their food choices may have consequences for their health (Goetzke et al. 2014), and the group of functional foods can offer interesting opportunities in this respect. Improving health through food is not a new idea. Already in ancient Greece, the doctor of medicine Hippocrates stated: “Let food be thy medicine and medicine be thy food”.

In recent years, food scientists have explored the field of angiotensin I‐converting enzyme (ACE) inhibitors from various food sources and food production side streams (e.g. reviewed by Vercruysse et al. 2005). Angiotensin I‐converting enzyme is central to the development of hypertension, which is a significant contributor to cardiovascular diseases and an increasing problem worldwide. Recent estimates show that approximately 25% of the world's adult population (25 years and over) suffer from hypertension (WHO, 2012).

In the past decade, studies have shown examples of peptides derived from meat that demonstrate antihypertensive activity (Ahhmed and Muguruma 2010). This opens up for the possibility of both producing designed meat products with health‐promoting properties and increasing the value of, for example, by‐products and other low‐value meat cuts.

Another issue that needs to be addressed is the fact that there is an increasing number of people worldwide, especially among the elderly, who suffer from protein deficiency. The challenge for this group is their, at times, decreased appetite. By adding proteins to, for example, meat products, the protein concentration increases substantially, and the elderly do not need to consume a greater volume of food, which is clearly an advantage. A very recent study on the preferences for protein‐enriched functional foods among the elderly showed that the elderly prefer protein‐enriched foods based on healthy products that they consume frequently (Van der Zanden et al. 2014).

Hydrolyzed proteins of animal origin are interesting as a source of peptides with bioactivity in relation to ACE inhibition. It is furthermore speculated that hydrolyzed proteins are more easily digested in the human body compared with the original protein. However, this still needs to be demonstrated. In the literature, enzymatic hydrolysis of protein‐rich materials is a process frequently used in the production of peptides. The degree of purification of the hydrolysates varies greatly. The hydrolysate can be used as a whole (Meinert et al. 2013) or as a single isolated component, for example, the peptide M6 (Muguruma et al. 2009).

When producing healthy foods, it is equally important that the eating quality is high, as otherwise people would most probably avoid these products despite their beneficial health effects. This might be a challenge with respect to hydrolysates in general, since the hydrolysates may result in chemical and/or bitter flavor characteristics (Meinert et al. 2013).

The aim of this study was to screen the applicability of hydrolysates as a source of peptides with ACE‐inhibitory activity and as a source of proteins. The application test was carried out using a traditional Danish meat product, saveloy. This is a well‐known Danish meat product, and, with minced meat as its basis, the hydrolysates can easily be evenly distributed. The saveloys were produced based on two recipes, one with pork and one with a mixture of pork and beef. Hydrolysates of different origin and in different concentrations were added to the saveloys. ACE‐inhibitory activity was measured and various chemical tests were performed. Furthermore, the sensory evaluations were performed by both a trained panel and elderly consumers (60+).

## Materials and Methods

### Experimental design

**Figure 1:**
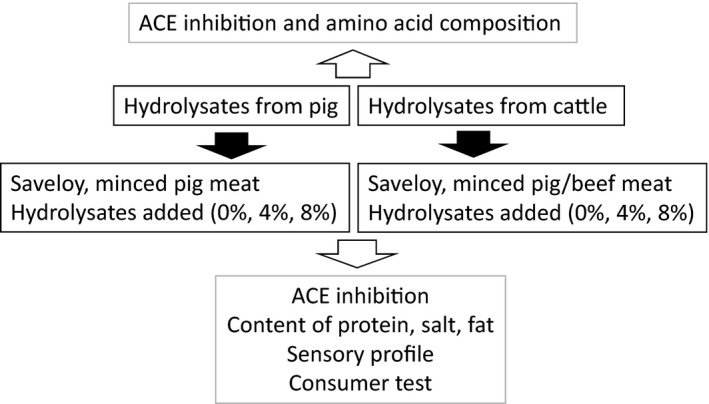
Experimental design. Analyses were performed on hydrolysates and saveloys with added hydrolysates. Two batches of saveloys were produced, one based solely on pig meat and one based on a mixture of pig and ox meat.

The overall experimental design is shown in Figure 1.

### Raw materials

Hydrolysates were produced from six different by‐products originating from pig and cattle, respectively. The source of by‐product was selected on the basis of a preliminary screening (data not shown). The selected by‐products were: porcine rind, porcine heart, porcine rectum, bovine hide, bovine heart, and bovine diaphragm. The by‐products were collected from the slaughterhouse on a random day to reflect the natural variation and availability of by‐products.

### Enzymatic hydrolysis

The hydrolysates were produced by heating the homogenized by‐products to 80°C for 2–10 h followed by fat separation. An enzyme mixture was added to the residual mass, and this was incubated for 1 h at 53–56°C. The hydrolysis process was stopped by heating the mixture to 90°C. The hydrolysates were concentrated and spray dried, either directly or after a 10,000 Dalton separation (ultrafiltration). The differences in hydrolysis are related to the differences in the raw materials.

### Production of saveloy

Saveloys were produced from minced pork or a mixture of minced pork and beef. The saveloys were processed based on a standardized industrial recipe. Ingredients used for the processing of saveloys included ice water, fat trimmings, pork trimmings, beef trimmings, jowl butt, potato flour, soy isolate, phosphate, a mixture of herbs, nitrite salt, dried onions, and ascorbic acid. Meat and trimmings were presalted with 1.5% nitrite salt (approx. 60 ppm nitrite) + 0.5% NaCl.

The meat and other ingredients, including hydrolysate, were mixed to form a forcemeat. The spray‐dried hydrolysates were added as an extra ingredient (4% and 8% weight) to the basic forcemeat without adjusting the other ingredients. A preproduction showed no functionality of the hydrolysates (data not shown). A control without added hydrolysate was produced for each of the two recipes. The temperature of the forcemeat was controlled by the gradual addition of ice water (approx. 10°C). The forcemeat was processed to the desired grind suitable for stuffing followed by thorough kneading (under vacuum).

The forcemeat was stuffed into 60 mm sterile casings, cooked at 89°C in a boiler cabinet until the saveloys reached a core temperature of 75°C. The saveloys were stored at 2°C until analysis. One batch, 12 saveloys, each weighing 1 kg, were produced. One separate saveloy was used for each analysis.

It is important to note, that hydrolyzed heart, rectum, and rind from pig was solely added to the saveloys based on pig meat. Likewise, hydrolyzed bovine heart, hide, and diaphragm were solely added to the saveloys based on a mixture of pig and beef.

### Screening of ACE inhibition

The inhibition of ACE was analyzed by spectrophotometric assay using slight modifications of the descriptions by Cushman and Cheung (1971), Nakamura et al. (1995) and Katayama et al. (2003). The analysis is based on the enzymatic conversion of hippuryl‐L‐histidyl‐L‐leucine (HHL) to hippuric acid (HA). The ACE used in the analysis was from rabbit lung.

Twelve *μ*L of the hydrolysate/minced saveloy dissolved in 0.1 mol/L borate buffer (dilution series, 12 concentration levels, mg/mL), was added to 100 *μ*L of the substrate solution (including HHL). The reaction was started by adding 40 *μ*L enzyme solution. The reaction mixture was incubated for 30 min. at 37°C, and the reaction was stopped by adding 1.100 *μ*L 0.1 mol/L HCl. A control was run without enzyme. The HA formed was extracted with 3.0 mL ethyl acetate, which was added to the sample while shaking vigorously followed by centrifugation for 10 min. Two mL of the ethyl acetate layer was transferred to a tube and dried using a vacuum evaporator at 45°C for 10 min. HA was redissolved in 2 mL 0.1 mol/L NaCl and the absorbance at 228 nm was measured in disposable cuvettes.

ACE‐inhibitory activity was calculated as follows:

Inhibition(%)=[(C−S)/(C−B)]×100

*S*: absorbance of sample; *C*: absorbance of control (buffer for sample); *B*: absorbance of blank (hydrochloric acid was added before ACE).

Inhibitory activity was expressed as IC_50_ (mg/mL) of the sample in an assay. An increase in IC_50_ indicates a decrease in ACE‐inhibitory activity.

### Amino acid composition in hydrolysates

The hydrolysates were analyzed for amino acid composition (single determination) by the laboratory under the Danish Veterinary and Food Administration. The method was based on Council regulation (EC). No 152 (2009) and ISO 13903 (2005). Lysine (EF/152/2009 AKK_424), methionine (EF/152/2009 AKK_424), cystine (EF/152/2009 AKK_424), threonine (EF/152/2009 AKK_424), proline (EF/152/2009), and valine (EF/152/2009), total content for water (EF/152/2009).

### Fat content of saveloys

The analysis of fat (determination in duplicate) is based on the principles of a gravimetric SBR method (NMKL, 1989). The method is accredited according to ISO 17025.

### Protein content of saveloys

The protein content (N × 6.25) was determined in duplicate as described in AOAC 981.10 (1983). The method is accredited according to ISO 17025. The Kjeldahl is an indirect measure of proteins. In this article, the term protein is used, even though the protein content in the saveloys with added hydrolysates is a mixture of various nitrogen‐containing compounds from meat and hydrolysates.

### Sodium chloride

The sodium chloride content was analyzed in duplicate in accordance with NMKL No. 178 (2004). The method is accredited according to ISO 17025.

### Sensory profile

The saveloys were evaluated using a sensory profile analysis in accordance with ASTM MNL No. 13 (1992), ISO 4121 (2003) and ISO 13299 (2003). The sensory profile analysis was performed by nine trained assessors.

The saveloys were assessed in two separate sessions, one session for each of the two recipes. Before each of the two sessions, a training session was conducted on selected saveloys. The aim of the training was to familiarize the assessors with the product and to achieve a common terminology. Fizz calculations and profile plots from Panelcheck were used during the training session to assess the performance of the assessors in terms of agreement and use of scales. During the assessor training, Fizz calculations (version 2.46B; Biosystems, Couternon, France) and profile plots from Panelcheck were used to adjust the panel.

The saveloys were sliced with a kitchen slicer to a thickness of 2 mm and the slices were served at a temperature of approx. 17°C. Each assessor received two slices. Samples were served in petri dishes with lids labeled with three‐digit codes. In order to minimize a carry‐over effect, the samples were randomized within each session. Ratings were performed using the software system FIZZ (Biosystems, FIZZ, Sensory software version 2.46B).

The sensory profile analysis was performed on seven different samples for each type of saveloy (pig based or pig/beef based), with two replicates. The intensity of the 14 attributes for pork saveloy and 17 attributes for the pork/beef saveloy was rated on an unstructured 15‐cm line scale ranging from 0 (no intensity) to 15 (high intensity).

### Consumer liking

The consumer segment 60+ was chosen in this study because the risk of hypertension increases with age (Danish Heart Foundation), and because this segment could generally benefit from an increased intake of proteins. Consumers aged 60+ rated the liking of the saveloys on a 15 cm unstructured line scale ranging from “like not at all” to “like very much”. Each consumer was served seven different saveloys, six with hydrolysate and one control. The pork saveloys and the pork/beef saveloys were evaluated on two different days. The saveloys were served with rye bread to reflect a real‐life eating situation. The consumer test was performed as a central location test at DMRI, Roskilde, Denmark.

### Statistical analyses

A PCA analysis was performed on the sensory data (profile) using the free software PanelCheck (ver. 1.4.0., Nofima, Norway) (www.panelcheck.com).

## Results and Discussion

### ACE‐inhibitory activity

The ACE activity of the hydrolysates was screened both in the pure powder and in the saveloy. The IC_50_ values are shown in Table 1, with 95% confidence interval estimated using inverse regression (Draper and Smith 1998).

**Table 1:** ACE‐inhibitory activity in hydrolysates and in saveloys (95% confidence intervals in brackets). The ACE activity in the two control saveloys was below 50% of inhibition, and the IC_50_ value could not be determined (“–”) 95%

Hydrolysate (raw material)	IC_50_ (mg hydrolysate/mL)	Saveloy (code)	IC_50_ (mg saveloy/mL)
Porcine rind	15 (14–17)	4%	146 (139–155)
		8%	65 (60–71)
Porcine heart	9.7 (9.0–10)	4%	309 (289–330)
		8%	147 (139–164)
Porcine rectum	7.0 (5.0–9.0)	4%	70 (60–80)
		8%	46 (45–48)
Bovine hide	60 (58–63)	4%	462 (427–519)
		8%	287 (270–307)
Bovine heart	9.2 (9.0–9.6)	4%	125 (114–136)
		8%	69 (47–82)
Bovine diaphragm	8.5 (8.3–8.8)	4%	118 (112–131)
		8%	52 (44–65)
		Control pork saveloy	–
		Control pork/beef saveloy	–

It can be seen from Table 1 that the saveloys without added hydrolysates do not display ACE‐inhibitory activity. By increasing the added amount of hydrolysates, the IC_50_ value decreased by a factor of 1.5–2, indicating an increase in inhibitory activity corresponding generally to the amount added.

### Amino acid content in hydrolysates

It has been reported that the ACE‐inhibitory activity may be particularly linked to proline‐rich peptides (e.g. Cushman and Ondetti 1991; Lanzer et al. 2007). It is known that the proline content is generally high in skin such as porcine rind and bovine hide. The content of selected amino acids in the hydrolysates can be seen in Table 2.

**Table 2:** Amino acid content (g/kg dry matter) and water content (%) in the hydrolysates. The high contents of proline in porcine rind and bovine hide are highlighted in bold

g/kg	Pig			Ox		
	Heart	Rectum	Rind	Hide	Heart	Diaphragm
Lysine	62.9 ± 4.537	49.2 ± 3.544	36.8 ± 2.655	37.6 ± 2.7	67.2 ± 4.845	59.6 ± 4.300
Methionine	15.5 ± 1.33	12.2 ± 1.04	6.91 ± 0.592	7.7 ± 0.7	15.7 ± 1.34	15.2 ± 1.30
Cystine	6.52 ± 0.548	6.62 ± 0.557	1.49 ± 0.125	0.6 ± 0.05	6.88 ± 0.579	5.12 ± 0.431
Threonine	29.8 ± 2.25	25.3 ± 1.92	17.4 ± 1.32	18.3 ± 1.4	35.0 ± 2.65	27.8 ± 2.11
Proline^a^	37.3	59	**124**	**139**	41.2	52.9
Valine	38.3 ± 2.76	30.4 ± 2.19	23.2 ± 1.67	22.9 ± 1.7	38.0 ± 2.74	32.2 ± 2.32
Water, %	4.8 ± 0.1	3.7 ± 0.1	4.2 ± 0.1	7.1 ± 0.2	4.6 ± 0.1	4.1 ± 0.1

The analysis for proline is not accredited and the uncertainty levels is not given.

Comparing the hydrolysates high in proline content (porcine rind, bovine hide) with the corresponding ACE‐inhibitory activity show no correlation. Indeed, bovine hide contains the highest amount of proline but display the lowest ACE‐inhibitory activity. This is a very simplified comparison not taking into account that the activity of proline is based on its position in the peptides. How proline is bound in the peptides or the basic nature of the peptides in the hydrolyzed bovine hide is not known. Therefore, it cannot readily seen from the amino acid content alone, whether a given hydrolysate has potential as ACE‐inhibitor or not.

### Saveloy quality

The quality of the saveloys was measured based on a number parameters in order to provide a thorough description of the sausages. Furthermore, these measures were conducted in order to ensure that variation in other factors than added hydrolysate was minimal.

The protein content has importance in relation to the nutrient value of the saveloys (see Table 3).

**Table 3:** The protein content of the two control saveloys and the saveloys with added hydrolysates

Saveloy (code)	N‐compounds (g/100 g)^a^
Control pig meat	11.6
Porcine rind 4%	14.7
Porcine rind 8%	17.9
Porcine heart 4%	14.1
Porcine heart 8%	16.7
Porcine rectum 4%	13.9
Porcine rectum 8%	16.6
Control pig/beef meat	10.5
Bovine hide 4%	14.8
Bovine hide 8%	18.3
Bovine heart 4%	13.8
Bovine heart 8%	16.3
Bovine diaphragm 4%	14.0
Bovine diaphragm 8%	16.8

a Expanded uncertainty: 3.1% rel.

It is clear from Table 3 that the protein content increased, not surprisingly, with the addition of hydrolysates and with increasing addition of hydrolysates. The protein content in the saveloys with 8% added hydrolysates ranged from 16.6% to 18.3%.

The content of salt in foods is important, and the authorities generally advise consumers to lower their daily intake of salt or, more precisely, their intake of sodium. Some hydrolysates have been shown to possess umami characteristics and thereby enhance the taste of salt without increasing the salt content. The salt content in all the saveloys, including the control, was on average 1.85 g/100 g (1.8–1.9). This shows that the addition of hydrolysates does not result in an increase in the salt content. The recipe was not optimized with regard to salt content, and therefore more work could be done in this area. However, this was found to be beyond the scope of the study.

### Fat content

The fat content was measured as a quality parameter to ensure the unity in the batches of saveloys. The average content was 18.9 g/100 g with a minimum of 17.0 g/100 g and a maximum of 22.6 g/100 g.

### Sensory description and consumer liking

**Figure 2:**
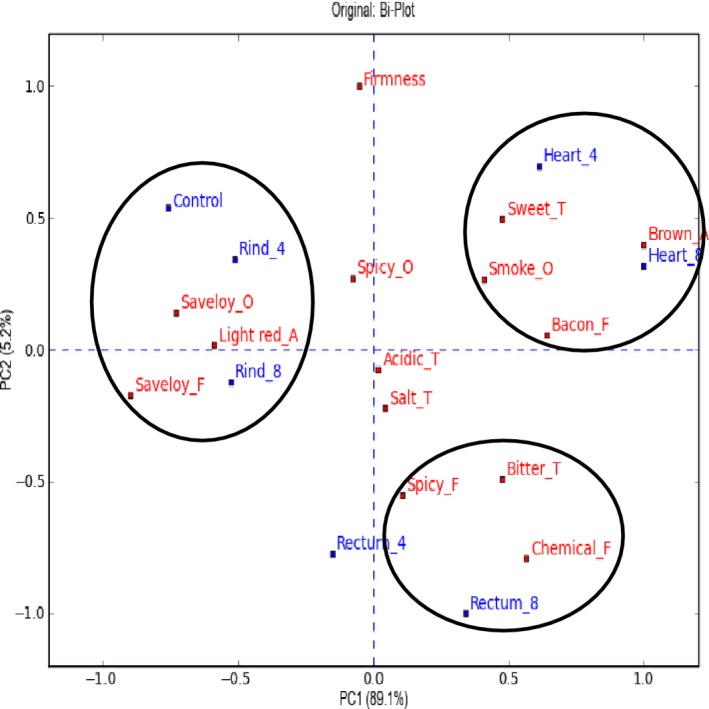
Biplot of the sensory profile data for saveloys made from pig meat. The saveloy code is “hydrolysate_added percentage”. The control is saveloy without added hydrolysate. F, flavor; T, taste; A, appearance; O, odor.

**Figure 3:**
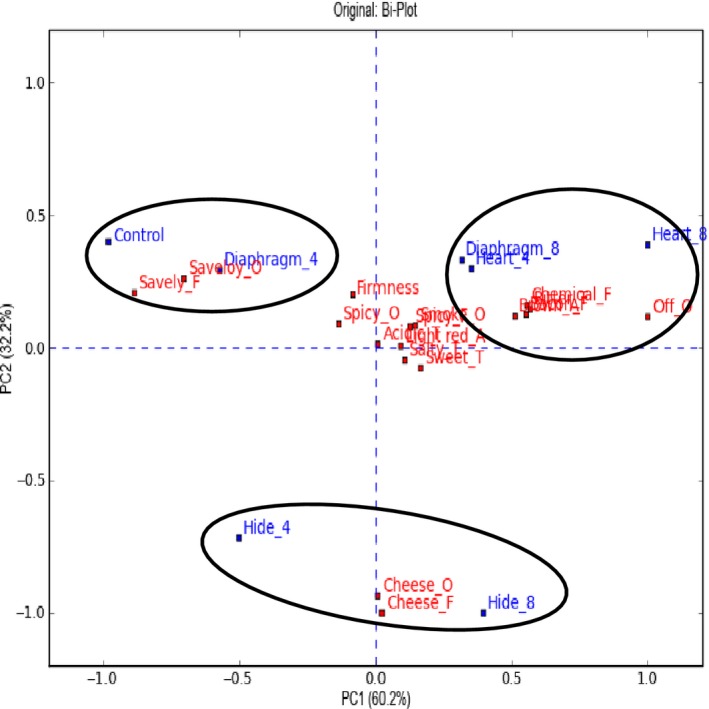
Biplot of the sensory profile data for saveloys made from a mixture of porcine and bovine meat. The saveloy code is “hydrolysate_added percentage”. The control is saveloy without added hydrolysate. F, flavor; T, taste; A, appearance; O, odor.

The sensory characteristics of the saveloys with added hydrolysates are important when evaluating the application potential of the hydrolysates. Each saveloy was described by a sensory profile (see Fig. 2 (saveloy with pork) and Fig. 3 (saveloy with pork and beef)).

The saveloys were divided into three groups related to the added hydrolysate.

From the biplot in Figure 2, it can be seen that the saveloys with added hydrolyzed rind are correlated with the control and with the saveloy flavor and odor attributes. This means that the hydrolyzed rind adds very little flavor to the saveloy and is very similar to the control. The saveloys with hydrolyzed rectum are correlated with chemical flavor, bitter taste, and spicy flavor. Finally, the saveloys with added hydrolyzed heart were described as sweet with a bacon‐like flavor. These saveloys were browner in appearance and had a smoke‐like odor.

The sensory profile analysis of the saveloys with pork and beef is shown in Figure 3. It can be seen that the saveloy with 4% added hydrolyzed diaphragm is similar to the control. The saveloys with added hydrolyzed bovine heart (4% and 8%) and the saveloy with 8% hydrolyzed diaphragm were described with the attributes “off‐odor”, “chemical flavor”, and “bitter taste”. The saveloy with bovine hide had a distinct flavor and odor of cheese.

The main reason for the differences in flavor of the saveloys can be ascribed to the nature of the hydrolysates, which are based on different raw materials. For example, pig heart taste different from pig rind. Furthermore, it seems that the distinct flavor of the hydrolysates increase in intensity with the increase in added amount. Based on the sensory characteristics, it is expected that the saveloys described with the attributes “chemical” and “bitter” might meet with resistance when presented to consumers.

### Consumer liking

Overall, the consumers did not rate the saveloys high with regard to liking, since the average liking score was 7–8 on a scale from 0 to 15. Looking more closely at the individual consumers, it can be seen that between 20% and 35% gave scores above 8. This can be interpreted as indicating that the consumers were somewhat neutral in their perception of the saveloys.

However, when looking at the comments and talking with the consumers after the tasting, it was clear that the low liking scores were mainly due to the absence of flavor rather than the presence of off‐flavors. Therefore, in this respect, the results provide a positive foundation for future product development.

## General Discussion

Saveloys were produced under industrial‐like settings, and even though the batch size of 12 kg is large for a pilot study, it is underlined that only one batch of each series of saveloys were produced. However, this study was a screening of the initial potential for application of the hydrolysates. Overall it was seen that standard saveloys were produced and therefore much of the seen variation can be ascribed to the addition of different hydrolysates. It can furthermore be seen that in relation to ACE‐inhibitory activity, all hydrolysates with the exception of bovine hide would be interesting to investigate further. One clear challenge is, however, the flavor which can be a limiting factor for the usage of the hydrolysates in meat products.

### The amount of saveloy to obtain an inhibitory effect?

In this study, saveloys were used as the carrier product partly due to the fact that they are based on minced meat, and also due to the ease with which the ingredients and hydrolysates can be mixed homogenously. Furthermore, saveloy is a very common and well‐known product in Denmark. The results showed that the ACE‐inhibitory activity in the hydrolysates was retrieved in the processed saveloys. However, one question remains unanswered: how much saveloy does one need to eat in order to obtain an inhibitory effect of ACE? This is an important question in relation to investigating the applicability of the hydrolysates as a substitute or supplement to medicine. The saveloys with the highest ACE‐inhibitory activity, 8% hydrolyzed porcine rectum (IC_50_ = 46), and 8% hydrolyzed bovine diaphragm (IC_50_ = 52), were used in the calculations. The IC_50_ values of the two saveloys were compared with Captopril, which is a known ACE inhibitor used in the medical treatment of hypertension. Thus, in order to obtain the same effect as a maintenance dose (25 mg Captopril), it will be necessary to consume about 250 g of saveloy with either 8% hydrolyzed porcine rectum or 8% hydrolyzed bovine diaphragm. On a daily basis, this is a rather large amount, also in relation to dietary recommendations for cold meat products. However, saveloy is just one type of product, and assuming that a wide range of products is available, it would support the accessibility and intake of the recommended dose on a daily basis. Another approach is to fractionate the hydrolysate and use the fraction containing the majority of the bioactive peptides. However, in this study it was decided to focus on the entire hydrolysate.

### The carrier product and the need for complex food matrices

Based on the ACE‐inhibitory activity measured in the processed saveloys, it was seen that saveloys with the highest ACE‐inhibitory activity were also characterized by the most intense off‐flavor (Figs. 2 and 3). This is indeed a challenge both in relation to gaining consumer acceptance and also finding a suitable carrier product to mask the off‐flavors, requiring carrier products with a more complex flavor matrix than saveloys. Saveloy is not necessarily the optimal carrier product, since the application of hydrolysates seems to be limited to approx. 8% due to the off‐flavor from some of the tested hydrolysates. Saveloy is characterized by a mild meat and light spicy flavor, whereas hydrolysates are characterized by a chemical sharpness and bitter flavor. Thus, saveloy may not have a flavor profile that is sufficiently complex to mask these off‐flavors. However, efforts were not made to optimize the products and the consumer reactions indicate that more spices could easily be added.

For further product development, a detailed characterization of the off‐flavors is needed in order to improve the development of carrier products and achieve an optimal flavor matrix, thereby masking the off‐flavors from the hydrolysates.

### Health claims of ACE‐inhibitory activity

A significant challenge associated with the promotion of ACE‐inhibitory compounds/hydrolysates is the requirement for scientific work, including clinical trials, to gain EFSA approval for health claims. EFSA has not yet approved the health effect of ACE‐inhibitory compounds due to lack of scientific documentation. The scientific substantiation and claimed effect of natural blood pressure support from bonito protein peptide has previously been evaluated based on EFSA (2010). EFSA did not find the scientific substantiation sufficient for the established cause and effect relationship between consumption of bonito protein peptide and maintenance of normal blood pressure, despite seven animal and in vitro studies, and sufficient characterization of the bonito protein peptide (EFSA, 2010). It therefore seems difficult to pursue a health claim for the time being.

### Protein enrichment

It was seen from the protein analysis (Table 3) that the addition of hydrolysates to the saveloys increase the total amount of protein, this can be a positive side effect. As reported in the Diogenes study by Larsen et al. (2010), a high‐protein diet based on lean meat, low‐fat dairy products, and beans combined with a low content of refined starches such as white bread and white rice improves the control of the glycemic index, resulting in improved maintenance of weight loss compared with a low‐protein diet.

Saveloys with 8% hydrolysate contain approx. 16 g protein/100 g compared with approx. 11 g/100 g in the control saveloys. The Nordic Nutrition Recommendations recommend a protein intake of 1.2 g protein/kg BW per day for the elderly. A person weighing 65 kg will need 78 g protein/day. Although the intake of saveloys alone should not cover the daily requirements, the development of new protein‐enriched versions of other well‐known and popular products targeting the elderly could be a future solution.

In this study, none of the consumers commented on the off‐flavor of the saveloys. This may indicate that the 60+ consumers were familiar with those saveloys, and therefore it is a suitable carrier product segment. In a younger consumer segment, the product acceptance may have been compromised by the perceived healthiness of the carrier product, since cold meat is sometimes referred to as an unhealthy product due to its salt content (Ares and Gámbaro 2007).

## Conclusion

Of the screened hydrolysates, porcine rectum and bovine diaphragm showed the highest ACE‐inhibitory activities. When added to saveloys, these two hydrolysates possessed the highest ACE‐inhibitory activities. Increasing the amount of added hydrolysate resulted in increased ACE‐inhibitory activity. The ACE‐inhibitory activities could not simply be explained by the amino acid profile. The content of nitrogen‐containing compounds in the saveloys increased with increasing addition of hydrolysate, with little difference between the added hydrolysates. The saveloys with added porcine rectum (8%), bovine diaphragm (8%), and bovine heart (4% and 8%) were assessed by the sensory panel as having the strongest off‐flavors (chemical flavor). No increase in salty taste resulting from the addition of hydrolysates was detected in the saveloys. Finally, the consumers found the saveloys too mild in flavor and recommended the addition of more spices.

## Conflict of Interest

None declared.
